# Case Report: Patient-derived organoids guiding dabrafenib–trametinib therapy in BRAF^V600E^-mutant metastatic gastric cancer

**DOI:** 10.3389/fonc.2026.1813652

**Published:** 2026-05-20

**Authors:** Caifeng Gong, Yang Yang, Qi Yan, Weiting Zhao, Minghao Zhang, Wen Zhang, Shuhui You, Ruihong Yin, Yang Huang, Yunshan Zhao, Aiping Zhou

**Affiliations:** 1 Department of Oncology, National Cancer Center/National Clinical Research Center for Cancer/Cancer Hospital, Chinese Academy of Medical Sciences and Peking Union Medical College, Beijing, China; 2 Darkjade Sciences, Inc., Beijing, China

**Keywords:** BRAF kinase inhibitor, organoids, preclinical model, stomach neoplasms, targeted therapy

## Abstract

**Background:**

BRAF^V600E^ mutations occur in approximately 0.4% of gastric cancer (GC) cases and are associated with aggressive disease and limited therapeutic options. Cytotoxic chemotherapy remains the standard first-line treatment for this rare molecular subtype, while evidence-based targeted strategies are lacking. Patient-derived organoids (PDOs) have emerged as a functional platform for precision oncology, but their clinical utility in BRAF^V600E^-mutant GC remains largely unexplored.

**Case presentation:**

A patient with rapidly progressive BRAF^V600E^-mutant metastatic GC presented with mediastinal invasion and malignant pleural effusion, posing a diagnostic challenge in distinguishing metastatic GC from a primary thoracic malignancy. Concordant histopathologic and genomic profiling across multiple tumor sites ultimately confirmed a gastric origin. Despite standard chemotherapy and immunotherapy, the disease progressed rapidly. Patient-derived organoids (PDOs) were established from malignant pleural effusion and used for functional drug sensitivity testing, which demonstrated marked sensitivity to dual BRAF-MEK inhibition. Based on these findings, the patient was treated with dabrafenib plus trametinib, resulting in prompt symptomatic improvement and radiologic regression of pleural effusion and ascites, consistent with PDO-predicted response.

**Conclusion:**

This case suggests that dual BRAF-MEK inhibition may represent a feasible precision treatment option for patients with BRAF^V600E^-mutant GC lacking established targeted therapies. Moreover, it highlights the potential role of PDOs as a functional decision-support platform for guiding individualized treatment selection in rare, genomically defined GC subtypes.

## Introduction

Gastric cancer (GC) remains a major cause of cancer-related mortality worldwide, particularly in East Asia, and outcomes for patients with advanced or metastatic disease remain poor (1). The incorporation of next-generation sequencing (NGS) into routine oncology practice has expanded opportunities for biomarker-driven therapy and reshaped the therapeutic landscape of multiple solid tumors. In GC, however, clinically actionable oncogenic drivers remain limited, especially among rare molecular subtypes. BRAF mutations represent one such uncommon alteration, occurring in approximately 2.2% of GC cases, with the BRAF^V600E^ substitution identified in only 0.4% (2, 3). To date, no established targeted therapeutic strategy has been defined for this rare molecular subtype; therefore, patients with BRAF^V600E^-mutant GC are generally managed according to standard systemic therapy paradigms for advanced GC, in which fluoropyrimidine-platinum combinations remain the backbone of first-line treatment (4, 5).

BRAF^V600E^ constitutively activates the mitogen-activated protein kinase (MAPK) pathway and represents a therapeutically actionable driver across multiple malignancies (6, 7). Accordingly, BRAF-targeted strategies, particularly combined BRAF-MEK inhibition, have demonstrated clinically meaningful activity in several BRAF^V600E^-driven solid tumors, including melanoma, non-small cell lung cancer (NSCLC), and thyroid cancer (8–10). Dabrafenib plus trametinib has therefore received tumor-agnostic approval for previously treated unresectable or metastatic solid tumors harboring BRAF^V600E^ mutations (11). However, the clinical role of BRAF-targeted therapy in GC remains largely unexplored. Here, we report a rare case of BRAF^V600E^-mutant GC that progressed after first-line chemotherapy and was subsequently treated with dabrafenib plus trametinib on the basis of PDO-guided drug sensitivity testing.

## Case description

**Figure 1:**
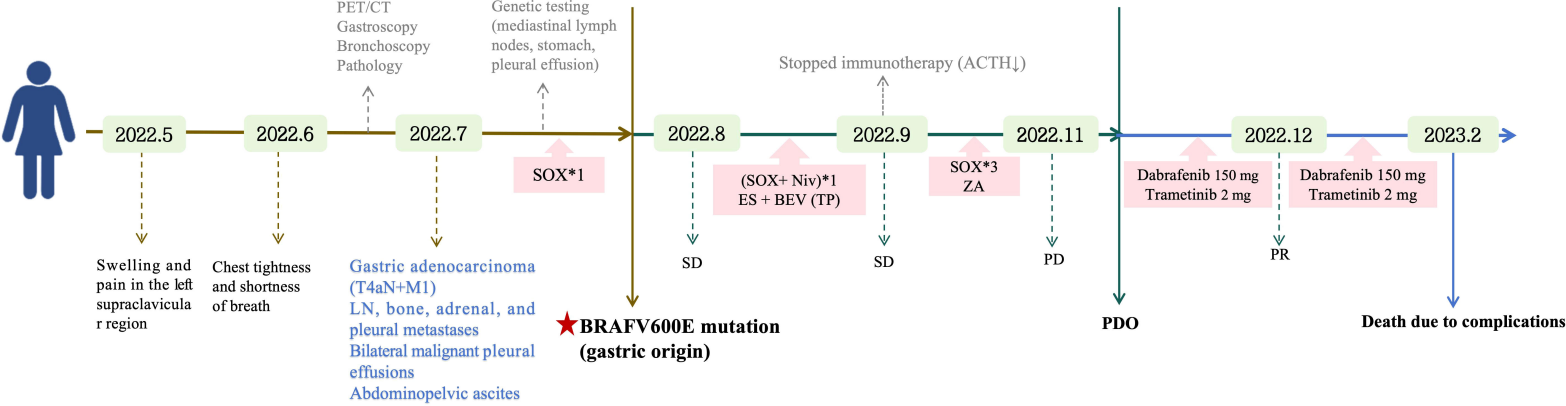
Clinical course and treatment timeline of the patient.

A 37-year-old woman with no family history of malignancy initially presented with left supraclavicular swelling and pain, which was treated as presumed subclavian vein thrombosis. Within one month, she developed progressive dyspnea. Subsequent evaluation revealed mediastinal lymphadenopathy and bilateral pleural effusions. Endoscopic biopsy and staging imaging confirmed stage IV poorly differentiated gastric adenocarcinoma with metastatic involvement of lymph nodes, bone, adrenal gland, and pleura, accompanied by malignant pleural effusion and abdominopelvic ascites. First-line SOX chemotherapy (oxaliplatin plus S-1) was initiated while molecular profiling was pending (**Figure 1**).

**Figure 2:**
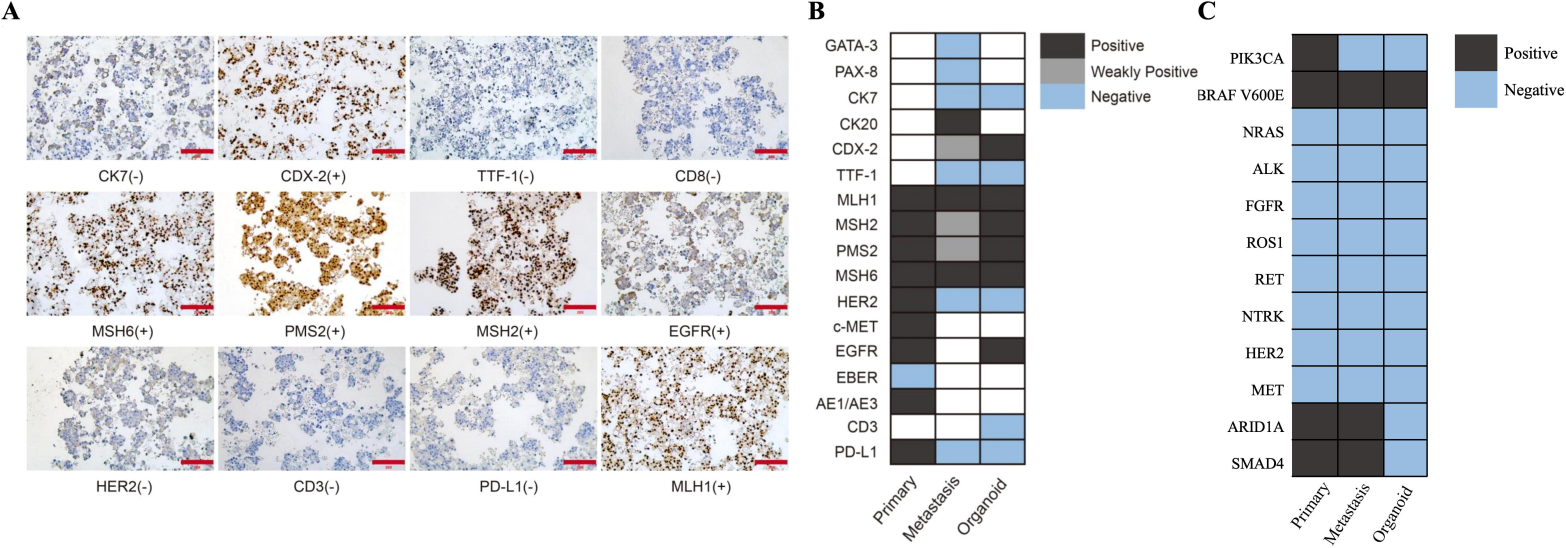
Histologic and molecular concordance between primary tumor, metastatic pleural effusion, and patient-derived organoids (PDOs). **(A)** Representative immunohistochemical staining demonstrating preserved tumor lineage markers, mismatch repair proteins, and immune-related markers in metastatic tissue and matched PDOs. **(B)** Heatmap showing concordant expression patterns of tumor-associated proteins between pleural effusion–derived samples and PDOs. **(C)** Genomic heatmap illustrating shared somatic alterations across the primary gastric tumor, metastatic pleural effusion, and corresponding PDOs, including consistent preservation of the BRAF^V600E^ mutation.

Next-generation sequencing (NGS) identified an oncogenic BRAF^V600E^ mutation, an alteration exceedingly rare in GC. Given the presence of lobar atelectasis on imaging and the established role of BRAF^V600E^ as a canonical driver in lung adenocarcinoma, an occult pulmonary primary was initially considered. To resolve this diagnostic uncertainty, NGS using the same sequencing panel was performed on both gastric biopsy and mediastinal lymph node specimens. All samples harbored an identical BRAF^V600E^ mutation with concordant co-mutation profiles, confirming a gastric origin of the mediastinal and pleural disease (**Figure 2C**).

Because of transient clinical and radiologic improvement, SOX chemotherapy was continued, with supportive treatment for pleural effusion and bone metastases. Given the patient’s HER2 1+ status and PD-L1 CPS of 1, nivolumab was introduced as part of first-line systemic treatment after discussion with the patient regarding the potential benefits and risks. This decision was made in accordance with the contemporary first-line treatment paradigm for advanced HER2-negative GC, in which PD-1 blockade may be combined with fluoropyrimidine-platinum chemotherapy (12). However, nivolumab was subsequently discontinued because of immune-related adrenal insufficiency. Despite minor tumor regression, bilateral pleural effusions persisted, and disease progression was documented after five cycles, corresponding to a progression-free interval of approximately three months (**Figure 1**).

At this point, therapeutic options were limited. Supported by previous studies demonstrating the feasibility of organoid generation from malignant body-fluid specimens in advanced GC (13), we established PDOs from malignant pleural effusion as an ex vivo platform to guide precision therapy. Immunohistochemical and molecular analyses demonstrated high phenotypic and genomic concordance among the primary tumor, metastatic lesions, and PDOs, including intact mismatch repair protein expression and concordant BRAF^V600E^ variant allele frequencies, supporting high model fidelity (**Figures 2A, B**).

On this basis, the PDO model was subsequently leveraged to functionally evaluate therapeutic sensitivity across multiple candidate regimens. PDOs were exposed to five dual-drug combinations comprising cytotoxic agents and/or targeted therapies, with oxaliplatin–5-fluorouracil (5-FU) serving as the reference control. These included dabrafenib combined with either trametinib or cetuximab, as well as apatinib paired with paclitaxel or irinotecan.

**Figure 3:**
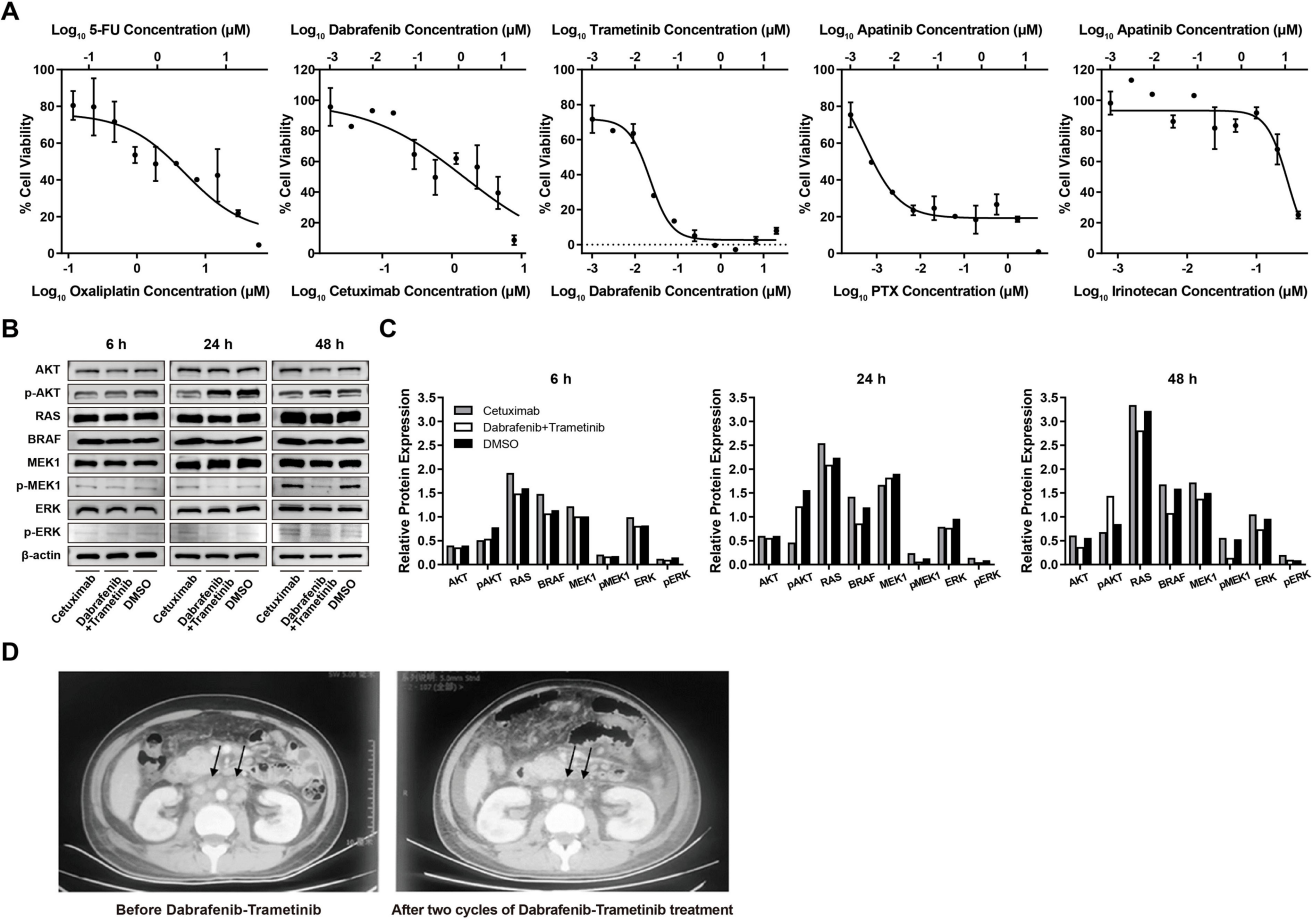
PDO-guided drug sensitivity and corresponding clinical response. **(A)** Dose–response curves from PDO-based drug screening demonstrating differential sensitivity across tested regimens. **(B)** Immunoblot analysis showing suppression of MAPK pathway signaling in PDOs following treatment with dabrafenib–trametinib compared with cetuximab or DMSO control at indicated time points. **(C)** Densitometric quantification of immunoblot signal intensities at 6 h, 24 h, and 48 h from a single experiment (n = 1). Reduced BRAF and phosphorylated MEK1 (pMEK1) expression was observed at 48 h following dabrafenib-trametinib treatment. No formal statistical comparison was applied. **(D)** Representative MRI images before and after dabrafenib–trametinib treatment, demonstrating marked reduction of ascites (black arrows).

PDO-based drug screening revealed marked heterogeneity in treatment response (**Figure 3A**). While oxaliplatin-5-FU, cetuximab-dabrafenib, and irinotecan-apatinib demonstrated only modest growth inhibition, dual BRAF-MEK blockade with dabrafenib-trametinib exhibited the most pronounced antiproliferative effect, with substantially lower IC_50_ values compared with standard chemotherapy. Given that BRAF^V600E^ drives constitutive activation of the MAPK pathway, dual BRAF-MEK inhibition was hypothesized to achieve maximal pathway suppression. Consistent with this mechanism, Western blot analysis from a representative experiment showed reduced phosphorylated MEK1 and total BRAF protein levels at 48 hours following dabrafenib-trametinib treatment, supporting suppression of MAPK signaling (**Figures 3B, C**).

Based on these findings and acceptable tolerability, the patient was treated with dabrafenib (150 mg twice daily) plus trametinib (2 mg once daily). Treatment resulted in rapid symptomatic improvement accompanied by marked radiologic reduction of pleural effusion and ascites (**Figure 2D**). However, despite this initial response, disease progression subsequently occurred, and the patient ultimately died from respiratory failure due to refractory malignant pleural effusion approximately six months after initiation of targeted therapy, highlighting the limited durability of response in the setting of advanced disease burden. Additional experimental details are provided in the Supporting Information.

## Discussion

Dabrafenib-trametinib dual inhibition is an established therapeutic strategy for BRAF^V600E^-driven malignancies, including melanoma, NSCLC, and thyroid carcinoma; however, its clinical efficacy in BRAF^V600E^-mutant GC remains poorly defined. In the present case, the patient experienced rapid disease progression after first-line oxaliplatin-based chemotherapy. Guided by PDO-based drug sensitivity testing, treatment was switched to dabrafenib plus trametinib, resulting in rapid symptomatic improvement and marked reduction of pleural effusion and ascites, suggesting potential therapeutic activity in this rare molecular subtype.

With the rapid development of immunotherapy, immune checkpoint inhibitors have become an important component of systemic treatment for advanced GC. In particular, dMMR/MSI-H GC is especially responsive to immunotherapy-based treatment, including chemotherapy plus PD-1 blockade, while dual immune checkpoint blockade such as PD-1 plus CTLA-4 inhibition has also shown promising activity in this molecular subset (14, 15). More broadly, PD-1 inhibitor plus chemotherapy has been incorporated into first-line treatment paradigms for HER2-negative advanced GC, particularly in patients with higher PD-L1 expression (16, 17). In our case, the patient also received chemo-immunotherapy as part of first-line treatment; however, the disease ultimately progressed, suggesting that alternative precision-based strategies may still be needed in some patients with BRAF^V600E^-mutant GC. Importantly, previous studies have demonstrated that the biological dependency of BRAF^V600E^ signaling is highly tumor-context dependent (18–20). In CRC, feedback reactivation of the EGFR pathway limits the efficacy of BRAF-MEK inhibition, necessitating combined BRAF and EGFR blockade, as demonstrated in the BEACON (21, 22) and BREAKWATER (23, 24) trials. In contrast, PDO-based analyses in this case suggested that BRAF^V600E^-mutant GC may rely on distinct signaling dependencies. Dabrafenib-trametinib elicited markedly greater growth inhibition than standard oxaliplatin-based chemotherapy and effectively suppressed MAPK signaling. Although adaptive activation of the PI3K/AKT pathway was observed at later time points, the addition of the EGFR inhibitor cetuximab provided limited additional antiproliferative benefit, indicating minimal utility of triple blockade in this context.

This case provides preliminary clinical evidence that dabrafenib-trametinib may have therapeutic activity in BRAF^V600E^-mutant GC. Although rapid symptomatic and radiologic improvement was observed, disease progression ultimately occurred, resulting in death from refractory malignant pleural effusion. The limited durability of response likely reflects advanced disease with high tumor burden and the emergence of adaptive resistance mechanisms. In contrast to CRC, where EGFR-dependent feedback predominates, BRAF^V600E^-mutant GC may rely on more heterogeneous bypass signaling pathways.

This case highlights the value of PDOs as real-time translational platforms for recapitulating tumor-specific drug sensitivities and informing individualized treatment decisions. Prior organoid studies in BRAF^V600E^-driven CRC have demonstrated the clinical relevance of functional drug screening beyond standard therapies (25). Our findings extend this concept to GC, suggesting that PDO-guided functional testing may be particularly useful for therapeutic prioritization in rare, genomically defined tumor subtypes where evidence-based options are limited.

Emerging evidence suggests that MAPK pathway inhibition not only induces compensatory bypass signaling but also modulates tumor-immune interactions, providing a potential biological rationale for exploring combination strategies incorporating immune checkpoint blockade in RAS- or BRAF-driven tumors (26, 27). In GC, future studies should explore such combinations and integrate longitudinal ctDNA monitoring to better characterize treatment response and resistance dynamics (28).

However, several limitations of organoid-based drug sensitivity testing should be acknowledged. First, as a single-case report, our findings are hypothesis-generating and cannot establish the clinical efficacy of this strategy in BRAF^V600E^-mutant GC. Second, although the pleural effusion-derived PDO preserved key histologic and genomic features of the patient’s tumor, organoid models do not fully recapitulate the native tumor microenvironment, including stromal, immune, and vascular components, which may influence treatment response (29). Third, ex vivo drug sensitivity may not fully reflect *in vivo* pharmacokinetics, systemic drug exposure, or host-related factors affecting efficacy and tolerability (29). In addition, although pleural effusion provided a practical and informative source for PDO generation in this case, a model established from a single disease site and time point may not fully capture the complete spatial and temporal heterogeneity of advanced disease (30). Finally, culture-related variables, including matrix conditions and passage-associated changes, may also influence organoid behavior and drug-response readouts (31).

## Conclusions

This case suggests potential therapeutic activity of dabrafenib-trametinib in BRAF^V600E^-mutant GC and underscores the translational utility of PDO-based functional testing to guide treatment decisions in rare, genomically defined tumor subtypes.

## Data Availability

The raw data supporting the conclusions of this article will be made available by the authors, without undue reservation.

## Supplementary material

Supplementary Figure 1 Representative baseline diagnostic CT images at presentation. **(A–D)** Chest CT images demonstrating bilateral pleural effusions, mediastinal involvement, lobar atelectasis, and pleural metastatic disease. **(E–H)** Abdominal and pelvic CT images showing upper abdominal disease burden and abdominopelvic ascites, illustrating the initial extent of metastatic gastric cancer.
