# Supplementary figures and images for: Case Report: Patient-derived organoids guiding dabrafenib–trametinib therapy in BRAFV600E-mutant metastatic gastric cancer
Source: Front Oncol. 2026 May 20;16:1813652. doi: 10.3389/fonc.2026.1813652 (PMC13229687; doi:10.3389/fonc.2026.1813652)

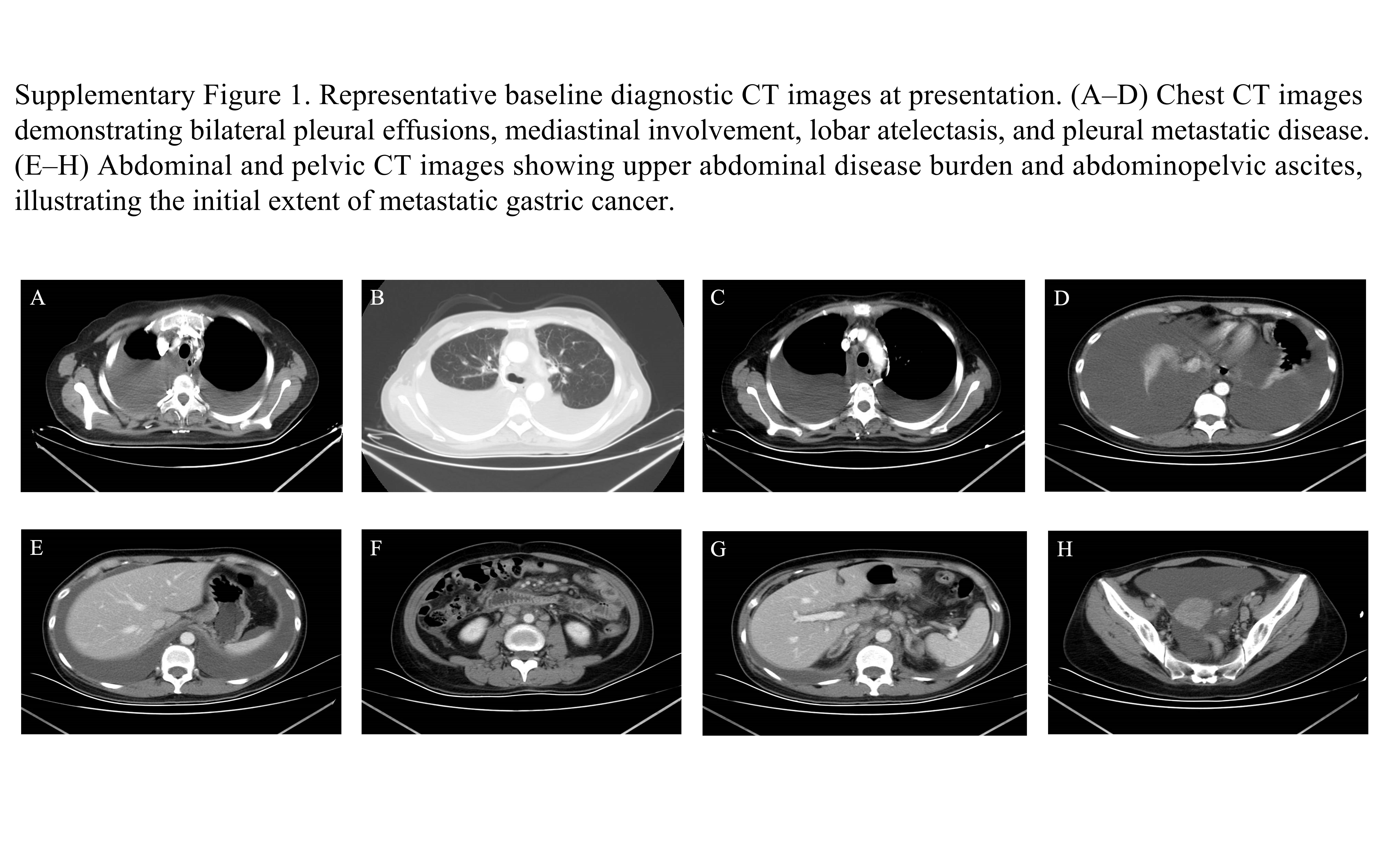

Supplement: Supplementary Figure 1 — Representative baseline diagnostic CT images at presentation. (A–D) Chest CT images demonstrating bilateral pleural effusions, mediastinal involvement, lobar atelectasis, and pleural metastatic disease. (E–H) Abdominal and pelvic CT images showing upper abdominal disease burden and abdominopelvic ascites, illustrating the initial extent of metastatic gastric cancer. [file Image1.jpeg]
