# Supplementary material for: Case Report: Patient-derived organoids guiding dabrafenib–trametinib therapy in BRAFV600E-mutant metastatic gastric cancer
Source: Front Oncol. 2026 May 20;16:1813652. doi: 10.3389/fonc.2026.1813652 (PMC13229687; doi:10.3389/fonc.2026.1813652)
Supplement: Supplementary file 2 [file DataSheet1.docx]

**Materials and methods**

***Informed consent.*** The cancer organoid culture was conducted using patient pleural effusion from a 37-year-old female. Complete written informed consent was obtained from the patient for the publication of this study.

***Organoid culture.*** The freshly extracted pleural effusion fluid was centrifuged at 500×g for 5–10 min to obtain tumor cell pellets. Erythrocytes were lysed at room temperature with red blood cell lysis buffer (Beyotime, #C3702). The cell pellets, which consisted mainly of tumor cells, were washed twice before being resuspended in cold GC organoid culture medium. Thereafter, Matrigel (Corning, #356231) was added to obtain a final concentration of 5%. The cell suspension mixture was immediately plated onto ultralow attachment 24-well plates (Corning, #3473) with precool tips and incubated at 37°C and 5% CO_2_ to allow Matrigel to gel for 30 min. Sufficient GC organoid culture medium was added to the cells, which were subsequently placed in an incubator for culture. The organoids tested negative for mycoplasma contamination and were passaged every 5–8 days when they had grown to an appropriate size (100–200 μm). Organoids were collected by centrifugation at 300×g for 3 min. After the supernatant was removed, 1–2 mL of TrypLE Express (Gibco, #12605028) was added to the cell pellets. The cells suspended in TrypLE were incubated at 37°C for 3 min, followed by pipetting several times to ensure complete digestion into small cell clusters. Small cell clusters collected by centrifugation were resuspended in GC organoid culture medium with 5% Matrigel and added to ultralow attachment 24-well plates for culture as described above.

***Histology and immunohistochemistry.*** Primary and metastatic tumor tissues and organoids derived from pleural effusion fluid were fixed in 4% paraformaldehyde, dehydrated, embedded in paraffin, and subjected to standard H&E staining. Immunohistochemical staining was performed via primary antibodies against CK7 (ZSGB-Bio Cat# ZA-0573, RRID:AB_2938647), CDX-2 (Aeonian Biotech Cat# AE00163, RRID:AB_2750609), TTF-1 (Quanhui International Cat# NCL-TTF-1, RRID:AB_3452008), CD8 (MabTag GmbH Cat# hCD8-01, RRID:AB_11131950), MSH6 (Aeonian Biotech Cat# AE00380, RRID:AB_2924705), PMS2 (BD Biosciences Cat# 556415, RRID:AB_396410), MSH2 (Spring Bioscience Cat# E17792, RRID:AB_11219316), EGFR (Aeonian Biotech Cat# AE00248, RRID:AB_2814960), HER2 (Aeonian Biotech Cat# AE00171, RRID:AB_2750958), CD3 (ZSGB-Bio Cat# ZM-0417, RRID:AB_2890105), PD-L1 (Maxim biotechnologies Cat# RMA-0732, RRID:AB_3076319). Images were captured using a Nikon Eclipse Ts2-FL.

***Western blot.*** Protein samples from organoid lysates were separated by sodium dodecyl sulfate-polyacrylamide gel electrophoresis (SDS-PAGE) and transferred onto nitrocellulose membranes (EMD Millipore). Membranes were blocked with 5% nonfat milk at room temperature for 2 h and incubated overnight with the indicated primary antibodies. After three washes, membranes were incubated with the corresponding secondary antibodies for 1 h at 37°C. Signals were detected using an enhanced chemiluminescence kit. Primary antibodies against AKT, p-AKT, RAS, BRAF, MEK1, p-MEK1, ERK, and p-ERK were obtained from Cell Signaling Technology. The β-actin antibody was obtained from Sigma. Densitometric results were expressed as the ratio of target protein intensity to β-actin. The Western blot assay was performed once, and densitometric quantification was derived from this single experiment; therefore, no formal statistical analysis was applied.

***Drug screening.*** Organoids were harvested and digested with TrypLE Express, counted and evenly plated in a 384-well **platform** (Thermo Fisher Scientific RRID:SCR_008452, Cat# 142762) with ~2000 cells contained in each well. Drugs were administered to the organoids after 48 h of incubation. Ten concentrations of oxaliplatin (MCE, HY-17371), 5-FU (MCE, HY-90006), cetuximab (MCE, HY-P9905), dabrafenib (MCE, HY-14660), irinotecan (MCE, HY-16562), apatinib (MCE, HY-13342), trametinib (MCE, HY-10999), and paclitaxel (MCE, HY-B0015) were added to the cells in set combinations. DMSO (MP, #196055) and staurosporine (MCE, HY-15141) were used as negative and positive controls, respectively. Cell viability was measured with a CellTiter-Glo^®^ Luminescent Cell Viability Assay Kit (Promega RRID:SCR_006724, Cat# G7573) 72 h after dosing. The data were analyzed via GraphPad Prism 9 (RRID:SCR_002798).

***Quantification and statistical analysis.*** All analyses were performed in a blinded manner such that histological analysis, protein analysis, drug testing, and data analysis staff were unaware of the genotype and mutation site. The statistical methods used are outlined in the respective figure legends. Statistical analyses were performed with Microsoft Excel (RRID:SCR_016137) and GraphPad Prism 9 (RRID:SCR_002798).
